# Quantum Blue Reduces the Severity of Woody Breast Myopathy via Modulation of Oxygen Homeostasis-Related Genes in Broiler Chickens

**DOI:** 10.3389/fphys.2019.01251

**Published:** 2019-10-01

**Authors:** Elizabeth Greene, Joshua Flees, Sina Dadgar, Barbara Mallmann, Sara Orlowski, Ahmed Dhamad, Samuel Rochell, Michael Kidd, Caroline Laurendon, Hayley Whitfield, Charles Brearley, Narasimhan Rajaram, Carrie Walk, Sami Dridi

**Affiliations:** ^1^Center of Excellence for Poultry Science, University of Arkansas, Fayetteville, AR, United States; ^2^Department of Biomedical Engineering, University of Arkansas, Fayetteville, AR, United States; ^3^School of Biological Sciences, University of East Anglia, Norwich, United Kingdom; ^4^AB Vista, Marlborough, United Kingdom

**Keywords:** quantum blue, woody breast, growth performance, hypoxia, oxygen-sensing genes

## Abstract

The incidence of woody breast (WB) is increasing on a global scale representing a significant welfare problem and economic burden to the poultry industry and for which there is no effective treatment due to its unknown etiology. In this study, using diffuse reflectance spectroscopy (DRS) coupled with iSTAT portable clinical analyzer, we provide evidence that the circulatory- and breast muscle-oxygen homeostasis is dysregulated [low oxygen and hemoglobin (HB) levels] in chickens with WB myopathy compared to healthy counterparts. Molecular analysis showed that blood HB subunit Mu (HBM), Zeta (HBZ), and hephaestin (HEPH) expression were significantly down regulated; however, the expression of the subunit rho of HB beta (HBBR) was upregulated in chicken with WB compared to healthy counterparts. The breast muscle HBBR, HBE, HBZ, and hypoxia-inducible factor prolyl hydroxylase 2 (PHD2) mRNA abundances were significantly down regulated in WB-affected compared to normal birds. The expression of HIF-1α at mRNA and protein levels was significantly induced in breasts of WB-affected compared to unaffected birds confirming a local hypoxic status. The phosphorylated levels of the upstream mediators AKT at Ser473 site, mTOR at Ser2481 site, and PI3K P85 at Tyr458 site, as well as their mRNA levels were significantly increased in breasts of WB-affected birds. In attempt to identify a nutritional strategy to reduce WB incidence, male broiler chicks (Cobb 500, *n* = 576) were randomly distributed into 48 floor pens and subjected to six treatments (12 birds/pen; 8 pens/treatment): a nutrient adequate control group (PC), the PC supplemented with 0.3% myo-inositol (PC + MI), a negative control (NC) deficient in available P and Ca by 0.15 and 0.16%, respectively, the NC fed with quantum blue (QB) at 500 (NC + 500 FTU), 1,000 (NC + 1,000 FTU), or 2,000 FTU/kg of feed (NC + 2,000 FTU). Although QB-enriched diets did not affect growth performances (FCR and FE), it did reduce the severity of WB by 5% compared to the PC diet. This effect is mediated by reversing the expression profile of oxygen homeostasis-related genes; i.e., significant down regulation of HBBR and upregulation of HBM, HBZ, and HEPH in blood, as well as a significant upregulation of HBA1, HBBR, HBE, HBZ, and PHD2 in breast muscle compared to the positive control.

## Introduction

Poultry production supports the livelihoods and food security of billions of people worldwide. However, it is facing several challenges from a steep projected increase in global demand for high-quality animal proteins and the need to solve the problem associated with high incidence of metabolic disorders such as woody breast (WB) myopathy, which has garnered tremendous attention the last few years. WB disorder is emerging on a global scale (Sihvo et al., 2014; Mudalal et al., 2015) and has been described as an extreme palpable stiffness of breast muscle and a myodegeneration within *pectoralis major* fillets (Petracci and Cavani, 2012). This phenotypic hardness of breast muscle is associated with varying degree of firmness, pale color, surface hemorrhaging, and white stripes. In severe cases of WB, an eminent ridge-like bulge on caudal area of fillet is present and, in some cases, a viscous fluid cover and/or petechial multifocal lesions on the fillet surface is observed (Sihvo et al., 2014). Histologic evidence indicated multifocal degeneration and necrosis of muscle tissue with infiltration of inflammatory and fat cells (Sihvo et al., 2014).

Although the etiology of the disorder is still not known, several elegant high-throughput transcriptomic and proteomics studies speculated that several potential factors including localized muscular hypoxia (Mutryn et al., 2015), oxidative stress, increased levels of intracellular calcium, and muscle fiber type switching (Soglia et al., 2016) could contribute to WB myopathy.

In addition to the animal well-being concern, the impact of WB myopathy on poultry meat quality has resulted in heavy economic loss (Kuttappan et al., 2016). In fact, severe WB has a significant negative impact on meat texture, protein content, and water-holding capacity, and thereby, on consumer acceptability and purchase (Kuttappan et al., 2012; Mudalal et al., 2014; Chatterjee et al., 2016; Tasoniero et al., 2016). There is, therefore, a critical need to define the molecular signature(s) involved in WB myopathy for subsequent development of mechanism-based (genetic, nutritional, and/or management) strategies to reduce WB incidence. In the present study, we provide evidence that the circulatory and breast muscle oxygen homeostasis is dysregulated along with the activation of hypoxic signaling pathways in chickens with WB myopathy. We also found that quantum blue (QB), which has been shown to enhance hematological parameters in channel catfish (Peatman and Beck, 2016), improves the expression of oxygen-sensing genes in blood and breast muscle and reduces the severity of WB disorder.

## Materials and Methods

### Animals, Diet, and Experimental Design

A total of 576 1-day-old male broiler chicks (Cobb 500) were weighed at day of hatch and randomly assigned to 48 floor pens in an environmentally controlled house. There were 12 birds/pen. Each pen was covered with clean pine wood shaving and equipped with separate feeders and water lines. Birds were given *ad libitum* access to clean water and feed for the duration of the study. The ambient temperature was gradually decreased from 32°C for days 1–3, 31°C for days 4–6, 29°C for days 7–10, 27°C for days 11–14, and 25°C thereafter. A relative humidity of ∼30–40% and a 23 h light/1 h dark cycles were also maintained until the end of the experiment. The environmental temperature and humidity were also continuously recorded in each pen using HOBO pro V2 data loggers (ONSET, MA, United States).

Birds were fed one of six dietary treatments in a complete randomized design. The diets were a nutrient adequate positive control (PC) diet formulated to meet Cobb 500 nutrition requirements. *Myo*-inositol (MI, Sigma–Aldrich, St. Louis, MO, United States) was added to the PC diet at 0.30% to create a second diet (PC + MI). The third diet was considered the negative control (NC) diet with a reduction of available phosphorus (avP) (Table 1), calcium, and sodium by 0.15, 0.16, or 0.03%, respectively. The NC diet was then supplemented with 500, 1,000, or 2,000 phytase units (FTU)/kg to create diets 4 (NC + 500 FTU), 5 (NC + 1,000 FTU), and 6 (NC + 2,000 FTU), respectively (Table 1). The phytase was QB (AB Vista, Marlborough, United Kingdom) with an expected activity of 5,000 FTU/g.

**TABLE 1:** Ingredient and nutrient composition of the experimental diets, as-is basis.

**Ingredient (%)**	**Starter phase**		**Grower phase**		**Finisher phase**	
			
	**Diet 1–2**	**Diet 3–6**	**Diet 1–2**	**Diet 3–6**	**Diet 1–2**	**Diet 3–6**
Corn	60.100	61.720	65.070	66.690	67.088	68.708
Soy bean meal (46%)	33.382	33.112	28.286	28.016	25.833	25.563
Poultry fat	2.473	1.899	2.821	2.248	3.616	3.042
Dicalcium phosphate	1.610	0.792	1.481	0.663	1.284	0.466
Limestone	1.015	1.130	0.981	1.096	0.919	1.034
Salt	0.355	0.282	0.359	0.285	0.361	0.288
Sodium bicarbonate	0.120	0.120	0.120	0.120	0.120	0.120
DL-Methionine	0.330	0.328	0.285	0.283	0.249	0.247
L-Lysine HCl	0.244	0.248	0.233	0.237	0.181	0.185
L-Threonine	0.102	0.102	0.096	0.096	0.082	0.082
Choline chloride (60%)	0.031	0.028	0.029	0.026	0.028	0.026
Vitamin premix^1^	0.100	0.100	0.100	0.100	0.100	0.100
Trace mineral premix^2^	0.100	0.100	0.100	0.100	0.100	0.100
Selenium premix^3^	0.020	0.020	0.020	0.020	0.020	0.020
Santoquin	0.020	0.020	0.020	0.020	0.020	0.020
**Calculated nutrients (%)**						
Dry matter	88.12	87.94	87.99	87.81	87.98	87.80
AMEn (kcal/kg)	3,035	3,035	3,108	3,108	3,180	3,180
Crude protein	21.20	21.20	19.10	19.10	18.00	18.00
AID Lys	1.18	1.18	1.05	1.05	0.95	0.95
AID Met	0.61	0.61	0.54	0.54	0.50	0.50
AID TSAA	0.89	0.89	0.80	0.80	0.74	0.74
AID Thr	0.77	0.77	0.69	0.69	0.65	0.65
AID Trp	0.22	0.22	0.19	0.19	0.18	0.18
AID Arg	1.27	1.27	1.12	1.12	1.05	1.05
AID Ile	0.79	0.79	0.71	0.70	0.66	0.66
AID Val	0.86	0.86	0.78	0.78	0.74	0.74
Total calcium	0.90	0.74	0.84	0.68	0.76	0.60
Total phosphorus	0.71	0.56	0.66	0.51	0.61	0.46
Available phosphorus	0.45	0.30	0.42	0.27	0.38	0.23
**Phytate phosphorus**						
Sodium	0.20	0.17	0.20	0.17	0.20	0.17
Potassium	0.89	0.88	0.80	0.80	0.75	0.75
Chloride	0.30	0.25	0.30	0.25	0.29	0.24
Magnesium	0.17	0.17	0.16	0.16	0.15	0.15
Copper	16.85	16.86	16.21	16.22	15.90	15.90
Selenium	0.20	0.20	0.20	0.20	0.20	0.20
Choline	1,750	1,750	1,650	1,650	1,600	1,600
Linoleic acid	1.17	1.20	1.27	1.30	1.31	1.34
**Analyzed nutrients (%)**						
Crude protein	21.75	21.00	18.90	18.65	18.75	18.70
Phytate phosphorus			0.22	0.22	0.22	0.22

*^1^Supplied per kilogram of diet: manganese, 100 mg; magnesium, 27 mg; zinc, 100 mg; iron, 50 mg; copper, 10 mg; iodine, 1 mg. ^2^Supplied per kilogram of diet: vitamin A, 30,863 IU; vitamin D_3_, 22,045 ICU; vitamin E, 220 IU; vitamin B_12_, 0.05 mg; menadione, 6.0 mg; riboflavin, 26 mg; *d*-pantothenic acid, 40 mg; thiamine, 6.2 mg; niacin, 154 mg; pyridoxine, 11 mg; folic acid, 3.5 mg; biotin, 0.33 mg. ^3^Supplied 0.12 mg of selenium per kg of diet.*

Dead or culled birds were recorded daily and feed intake (FI, individual and cumulative) was adjusted for the day the bird died. Body weight was recorded weekly and body weight gain, feed conversion ratio (FCR, which measures the efficiency of the bird to convert feed into meat and expressed as kg feed/kg gain), and feed efficiency (FE, which is the inverse of FCR) were determined as previously described (Washburn et al., 1975).

The present study was conducted in accordance with the recommendations in the guide for the care and use of laboratory animals of the National Institutes of Health and the protocols were approved by the University of Arkansas Animal Care and Use Committee under Protocol No. 16084.

### WB Palpation and Scoring

As previously described (Mallmann et al., 2017), WB occurrence was estimated via live-bird palpation on a weekly basis. After slaughter process at d56, breast filets were macroscopically scored and classified to WB categories to the degree: 0, normal (NORM); 0.5–1.5, moderate (MOD) with mild hardening in the caudal S1 area; and 2–3, severe (SEV) with severe hardening and hemorrhagic lesions in the S1 region.

### Blood Sampling

For plasma samples, bloods were collected from eight birds/treatment in vacutainer tubes with plasma separation tube (PST) gel and lithium heparin and after centrifugation (1,500 × *g*; 10 min; 4°C), plasma was separated and stored at −20°C for later analyses of circulating metabolites and MI. For molecular target analysis, bloods were collected in tubes containing TRIzol LS reagent according to manufacturer’s recommendations (Life Technologies Corporation, CA, United States). Breast muscle samples were also collected as we previously described for molecular analyses (Orlowski et al., 2018). The remaining chickens were processed at the processing plant and carcass traits and meat quality were assessed.

### Circulating and Breast Muscle Myo-Inositol Measurement

Tissue (50–100 mg frozen weight) was homogenized in 1 ml of ice-cold 5% w/v (0.83 N) perchloric acid, 20 mM EDTA, Na_2_, in pyrex tubes with a IKA (Germany) T10 ULTRA-TURRAX^®^ homogenizer fitted with a S10N-8G-ST probe. The homogenate was held on ice for 15 min and centrifuged at 15,000 × *g* for 10 min at 4°C. The supernatant was diluted 50-fold in 18.2 mOhm cm water before analysis by HPLC-pulsed amperometry on an Antec (Netherlands) Carbohydrate Analyser fitted with a 3-mm diameter gold HyRef electrode. Chromatography of inositol followed the gradient and column conditions of Lee et al. (2018). A linear calibration curve with *r* > 0.995 was obtained with a six-point calibration curve of 0–5 μM inositol, 5 μl samples, and standards were injected. Plasma inositol was measured by the same method after treatment of 1 volume of plasma with 2 volumes of ice-cold 1 N perchloric acid to precipitate protein.

### Circulating Metabolite Measurement

As we previously described (Nguyen et al., 2015), commercial colorimetric diagnostic kits were used to measure plasma glucose (Ciba Corning Diagnostics Corp., OH, United States), triglycerides, cholesterol, and creatine kinase (CK, Chiron Diagnostics, Cergy Pontoise, France), lactate dehydrogenase (LDH, Bayer Healthcare, Dublin, Ireland), non-esterified fatty acids (NEFA, Wako Diagnostics, Mountain View, CA, United States), and uric acid (UA) levels (Pointe Scientific Inc., Canton, MI, United States) with an automated spectrophotometer according to manufacturer’s recommendations. Plasma total proteins were measured using Pierce BCA protein Assay kit (ThermoFisher Scientific, Rockford, IL, United States).

### Blood Chemistry, Gases, and Hematology

Blood pH, partial pressure of CO_2_ (pCO_2_), total CO_2_ (TCO_2_), partial pressure of O_2_ (pO_2_), bicarbonate (${\text{HCO}}_{3}^{-}$), base excess (BE), O_2_ saturation (sO_2_), sodium (Na), potassium (K), ionized calcium (iCa), glucose, hematocrit (Hct), and hemoglobin (HB) were determined using i-STAT Alinity system (SN:801128; software version JAMS 80.A.1/CLEW D36; Abaxis, Union City, CA, United States) with the i-STAT CG8 + cartridge test (ABBT-03P77-25) according to manufacturer’s recommendation. Before use, cartridges were allowed to equilibrate to room temperature overnight. Analysis was performed at room temperature using the temperature correction function of the i-STAT Alinity system. The i-STAT system was validated in many species including mammals (Stockard et al., 2007) and birds (Martin et al., 2010; Schaal et al., 2016).

### Diffuse Reflectance Spectroscopic Measurement of Oxygen Homeostasis in Breast Muscle

The optical spectroscopy instrument has been reported in detail previously (Dadgar et al., 2018). Briefly, the instrument consists of a halogen lamp (HL-2000, Ocean Optics, Dunedin, FL, United States), for illumination, a USB portable spectrometer (Flame, Ocean Optics, Dunedin, FL, United States), and a hand-held bifurcated fiber optic probe for light delivery and collection. The probe head that is placed in contact with tissue is 6.5 mm in diameter and consists of four illumination optical fibers (diameter = 200 μm; numerical aperture = 0.22) located at the center of the metal ferrule, and five detection fibers located at a source–detector separation distance (SDSD) of 2.25 mm away from the center (FiberTech Optica, ON, Canada). Diffusely reflected light from the chicken breast was collected in the spectral range of 475–600 nm by gently placing the probe in contact with the breast muscle. We have determined the penetration depth of this probe at SDSD of 2.25 mm to be ∼1.8 mm, based on established methods (Nichols et al., 2012). Spectra were collected with a custom LabVIEW (National Instruments, Austin, TX, United States) software controlled by a foot pedal with an integration time of 100 ms. From each animal, several spectra were measured from WB (caudal S1 region) and three contralateral normal sites (S2, S3, and S4) and averaged optical properties were used to represent that site. Spectra were background-subtracted to eliminate ambient light. This background-subtracted light was calibrated for light throughput by dividing it by background-subtracted reflected light intensity of an 80% reflectance standard (SRS-80-010; Labsphere, North Sutton, NH, United States).

A lookup table (LUT) (Rajaram et al., 2008)-based inverse model was used to fit the acquired optical data and extract wavelength-dependent absorption and scattering properties from tissue. To fit the model to the data, we limited scattering to follow a power-law dependence on wavelength, as described by Mourant et al. (1997), as following: ${\text{μ}}_{\text{s}}^{{}^{\prime }}\,\left(\text{λ}\right)={\text{μ}}_{\text{s}}^{{}^{\prime }}\,\left({\text{λ}}_{0}\right).{\left(\text{λ}/{\text{λ}}_{0}\right)}^{-\text{B}}$, where λ_0_ = 600*nm*. We assumed only oxygenated HB (HbO_2_), deoxygenated HB (dHb), and melanin to be the primary absorbers in spectral range of 475–600 nm and hence calculated μ_a_ as sum of the absorbing chromophores as: μ_a_(λ) = [Hb][ασHbO_2_(λ) + (1-α)σ_dHb_(λ)] + [Ml]mel(λ), where [Hb] and [Ml], respectively, are total HB (THB) and melanin concentrations. Alpha (α) is the oxygen saturation which represents the ratio of oxygenated (HbO_2_) to THB concentration [Hb]. The fixed absorption parameters, extinction coefficients of oxygenated HB (σHbO_2_), deoxygenated HB (σ_dHb_), and melanin (mel) were obtained from an online database^1^. LUT data generation and data analysis was performed in MATLAB (Mathworks, Natick, MA, United States).

### Reverse Transcription and Real-Time Quantitative PCR

**FIGURE 1:**
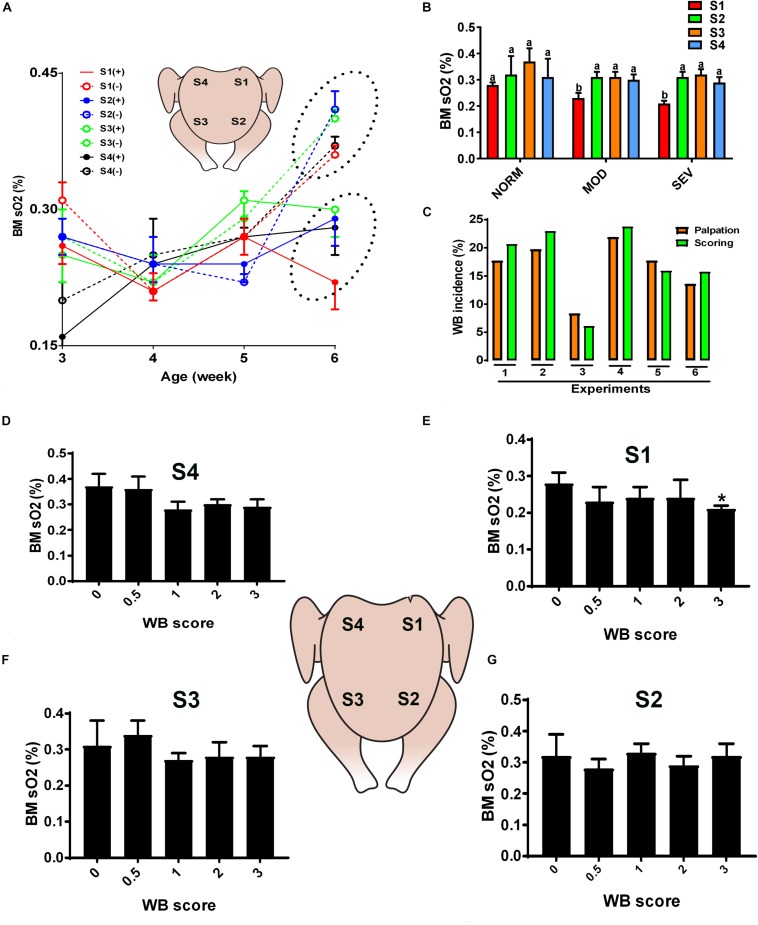
Dysregulation of oxygen levels in the breast muscle of WB-affected broilers. DRS measurement shows a significant lower sO_2_ levels in WB-affected birds compared to their healthy counterparts at 6 weeks of age, with higher magnitude in caudal S1 region **(A)**. Decrease of oxygen levels in MOD and SEV woody breast **(B)**. Correlation between palpation and scoring system **(C)**. Decrease of oxygen levels in SEV WB with score 3 in broiler breast muscle **(D–G)**. Data are presented as mean ± SEM (*n* = 50/group). ^∗^ and different letters indicate significant difference at *P* < 0.05. (+) WB-affected birds, (–) non-affected birds.

Breast muscle samples were collected from caudal S1 region (C) of unaffected birds and from S1 (WW, woody beast area) and S2 (WN, apparent healthy area) of WB-affected birds (Figure 1). Total RNA was extracted from chicken blood and breast muscle samples by using TRIzol LS (for blood) and TRIzol (for muscle) reagent (Life Technologies Corporation, NY, United States) according to manufacturer’s recommendations. RNA integrity and quality was assessed using 1% agarose gel electrophoresis and RNA concentrations and purity were determined for each sample by Take 3 Micro-Volume Plate using Synergy HT multi-mode micro plate reader (BioTek, Winooski, VT, United States). The RNA samples were RQ1 RNase-free DNase treated (Promega, WI, United States) and 1 μg RNA was reverse transcribed using qScript cDNA Synthesis Kit (Quanta Biosciences, Gaithersburg, MD, United States). The RT reaction was performed at 42°C for 30 min followed by an incubation at 85°C for 5 min. Real-time quantitative PCR (Applied Biosystems 7500 Real-Time PCR System) was performed using 5 μl of 10× diluted cDNA, 0.5 μM of each forward and reverse specific primer, and SYBR Green Master Mix (ThermoFisher Scientific, Rockford, IL, United States) in a total 20 μl reaction. Oligonucleotide primers used for chicken HB subunits and oxygen-sensing genes are summarized in Table 2. The qPCR cycling conditions were 50°C for 2 min, 95°C for 10 min followed by 40 cycles of a two-step amplification program (95°C for 15 s and 58°C for 1 min). At the end of the amplification, melting curve analysis was applied using the dissociation protocol from the Sequence Detection system to exclude contamination with unspecific PCR products. The PCR products were also confirmed by 2% agarose gel and showed only one specific band of the predicted size. For NCs, no cDNA templates were used in the qPCR and verified by the absence of gel-detected bands. Relative expressions of target genes were normalized to the expression of 18S rRNA and calculated by the 2^–ΔΔCt^ method (Schmittgen and Livak, 2008). Healthy birds and PC diet-fed birds were used as calibrators.

**TABLE 2:** Oligonucleotide real-time qPCR primers.

**Gene**	**Accession number^a^**	**Primer sequence (5′→3′)**	**Orientation**	**Product size (bp)**
*HBA1*	NM_001004376	TCCATGCTTCCCTGGACAA	Forward	59
		GTACTTGGCGGTCAGCACAGT	Reverse	
*HBBR*	NM_001004390	CCGAGGAGAAGCAGCTCATC	Forward	65
		TTCGGCACCGCATTCC	Reverse	
*HBM*	NM_001004375	GAGCAACCTGCATGCCTACA	Forward	59
		GCGACAACAGCTTGAAATTGAC	Reverse	
*HBZ*	NM_001004374	TGCCGTGACCACCATCTG	Forward	56
		CCAGCCCAATGGACTCAATC	Reverse	
*HBE*	NM_001081704	TCCTGCCTGCCAATTTGC	Forward	55
		CAGAGCATGAGCCACAACGT	Reverse	
*FPN1*	BM486402	CGCATAAGGCTAGCGCTTTC	Forward	62
		GTGTTGCCTTCCCCGACTT	Reverse	
*FTH1*	NM_205086	CCACGAGGAGCGTGAACAT	Forward	58
		TCCACCCCTCTGGTTTTGC	Reverse	
*FTL*	NM_204383	TGCTGGAGCTCGCCTACAG	Forward	60
		CCACGTGTGACTGATCAAAATATTC	Reverse	
*HEPH*	XM_420165	GGACTGGAATTATGCTCCAACAG	Forward	68
		CCTTTAGGCTACGTGTGATGCTT	Reverse	
*HJV*	XM_025143560	GCTCCGGATCACCAAAGCT	Forward	61
		AGCGGAACGTCTTCTCGTAGTC	Reverse	
*MB*	NM_00116775	GGCAGCACTTGAGACCTATCTATCT	Forward	59
		TCGCTGAGCCCCATGGT	Reverse	
*TFR2*	NM_205256	ACCTTGGAACTGGAGACCCTTAC	Forward	64
		GGTGGAAACTGGGTGTGGTT	Reverse	
*HIFPH2*	XM_015284393	CGCCGCAACCCTCATG	Forward	64
		AATACCACACTGTTATTGCGTACCTT	reverse	
*Akt*	AF039943	TTCAACGGTGATCTTTTGACTGA	Forward	64
		CGGGAATGTCTCTTGGTGGAT	Reverse	
*HIF*-1 α	NM_204297	AACACACCATGATATGTTCACGAAA	Forward	83
		CCCAGACGTAGCCACCTTGT	Reverse	
*PI3K*α	NM_001004410	GCCATCTTACTCCAGGCGTATC	Forward	70
		GAGGGACTTGGCTGTAGCTTCTC	Reverse	
*18S*	AF173612	TCCCCTCCCGTTACTTGGAT	Forward	60
		GCGCTCGTCGGCATGTA	Reverse	

*^*a*^Accession number refers to GenBank (NCBI). AKT, V-Akt murine thymoma viral oncogene homolog or protein kinase B (PKB); HBA1, hemoglobin subunit alpha 1; HBE, hemoglobin subunit epsilon; HBBR, hemoglobin beta, subunit rho; HBM, hemoglobin subunit mu; HBZ, hemoglobin subunit zeta; HEPH, hephaestin; HIF-1α, hypoxia inducible factor 1 alpha; HIFPH2, hypoxia-inducible factor prolyl hydroxylase 2; HJV, hemojuvelin; FTH1, ferritin heavy chain 1; FPN1, ferroportin 1; FTL, ferritin light chain; MB, myoglobin; PI3K, phosphatidylinositol 3-kinase; TFR2, transferrin receptor 2.*

### Conventional and Fluorescent Western Blot Analysis

Conventional immunoblot for breast muscle tissues was performed as we described previously (Flees et al., 2017; Nguyen et al., 2017). The rabbit polyclonal anti-HIF-1α (# LS-C287203, LSBio, Seattle, WA, United States), anti-phospho mTOR ser2481 (#2974), anti-mTOR (#2972), anti-phospho-PI3K P85tyr458 (#4228), and anti-PI3K (#3358) were used. Antibodies were purchased from Cell Signaling Technology (Danvers, MA, United States). Protein loading was assessed by immunoblotting with the use of rabbit anti-GAPDH (#sc-25778, Santa Cruz Biotechnology Inc., Dallas, TX, United States). Pre-stained molecular weight marker (Precision Plus Protein Dual Color) was used as a standard (BioRad, Hercules, CA, United States). The secondary antibodies were used (1:5,000) for 1 h at room temperature. The signal was visualized by enhanced chemiluminescence (ECL plus; GE Healthcare Bio-Sciences, Buckinghamshire, United Kingdom) and captured by FluorChem M MultiFluor System (Proteinsimple, Santa Clara, CA, United States). Image acquisition and analysis were performed by AlphaView software (Version 3.4.0, 1993-2011, Proteinsimple, Santa Clara, CA, United States).

For the fluorescent western blot analysis, 100 mg breast muscle tissue was homogenized using an IKA (Germany) T10 ULTRA-TURRAX^®^ homogenizer, fitted with a S10N-8G-ST probe, in 1 mL ice-cold radioimmunoprecipitaion assay (RIPA) buffer with Pierce phosphatase and protease inhibitors (Life Technology Corporation, NY, United States). The homogenate was held on ice for 15 min, centrifuged at 15,000 × *g* for 20 min at 4°C and the protein content of the supernatant was quantified by a Bradford assay (Life Technology Corporation, NY, United States). Protein (60 μg total) was resolved on a Sigma TruPAGE 4–12% gel. Samples were transferred to an iBlot 2 nitrocellulose membrane (Invitrogen, Life Technology Corporation, NY, United States) using an iBlot 2 transfer device (Life Technology Corporation, NY, United States). The membrane was incubated in 20 mL 5% goat serum (Merck, NJ, United States) in tris buffered saline with Tween 20 (TBST) for 1 h, then incubated with 1/1,000 dilution of primary rabbit polyclonal anti-Phospho-Akt (Ser473) or Akt (pan) antibody (Cell Signaling Technology #4060 or #4691, respectively, Danvers, MA, United States) and anti-β actin (#ab14128, Abcam, Cambridge, MA, United States) in 10 mL 5% goat serum in TBST overnight at 4°C. Subsequently, the membrane was washed three times with TBST for 10 min then incubated with 1/10,000 secondary antibody Goat Anti-Rabbit IgG H&L (Alexa Fluor 790, #ab186697) (Abcam, Cambridge, MA, United States) in 10 mL 5% goat serum in TBST at room temperature for 1 h. The membrane was washed three times with TBST for 10 min and imaged on a LI-COR Odyssey infrared imaging system. The membrane was then stained and imaged for total protein using amido black. Data were analyzed using the LI-COR Image Studio software, and normalized using total protein.

### Statistical Analysis

Data were analyzed as a completely randomized one-way ANOVA using the fit model platform in JMP Pro v 14.0 (SAS Institute, Cary, NC, United States). The model included diet. When diet was significant, means were separated using non-orthogonal contrast statements and *post hoc* Scheffe’s adjustment to reduce the likelihood of making a type-I error. Pen was considered the experimental unit for growth performance and carcass parameters. WB scores were analyzed as completely randomized one-way ANOVA using the categorical platform in JMP Pro v 14.0 (SAS Institute, Cary, NC, United States). Bird was the experimental unit and score was considered an ordinal variable. The model included diet. When diet was significant, score means between diets were separated using Pearson Chi-square. Differences between the frequency of each score within diet were also determined using Fisher’s exact test. Significance was accepted at *P* < 0.05. Gene and protein expression data were analyzed by Student “*t*”-test or one-way ANOVA when appropriate. If ANOVA revealed significant effects, the means were compared by Tukey’s multiple range test using the Graph Pad Prism version 6.00 for Windows (Graph Pad Software, La Jolla, CA, United States), and differences were considered significant at *P* < 0.05.

## Results

### The Circulatory- and Breast Muscle-Oxygen Homeostasis Is Dysregulated in Chickens With WB Myopathy

Quantification of optical properties using the diffuse reflectance spectroscopy (DRS) spectra and their LUT fits, in combination with palpation system, showed an age-dependent increase of WB incidence (data not shown) and an age-dependent increase of sO_2_ levels in normal breast muscle. However, the breast sO_2_ levels in WB-affected birds remained unchanged with age and were significantly lower compared to that of non-affected birds at 6 weeks of age (Figure 1A), with a significant higher magnitude in the affected caudal S1 region (Figure 1A). Further in depth analysis revealed a significant decrease of sO_2_ levels in S1 area of MOD and SEV WB compared to NORM breast (Figure 1B), indicating a poor oxygenation in MOD and SEV WB. Figure 1C illustrated a low variation (<2–3%) between the palpation and scoring system. When using a scoring scale of 0.5, severe WB with score 3 in caudal S1 region manifested significant low sO_2_ levels compared to the other scores; however S2, S3, and S4 regions did not elicit any significant differences between all the WB scores (Figures 1D–G).

**FIGURE 2:**
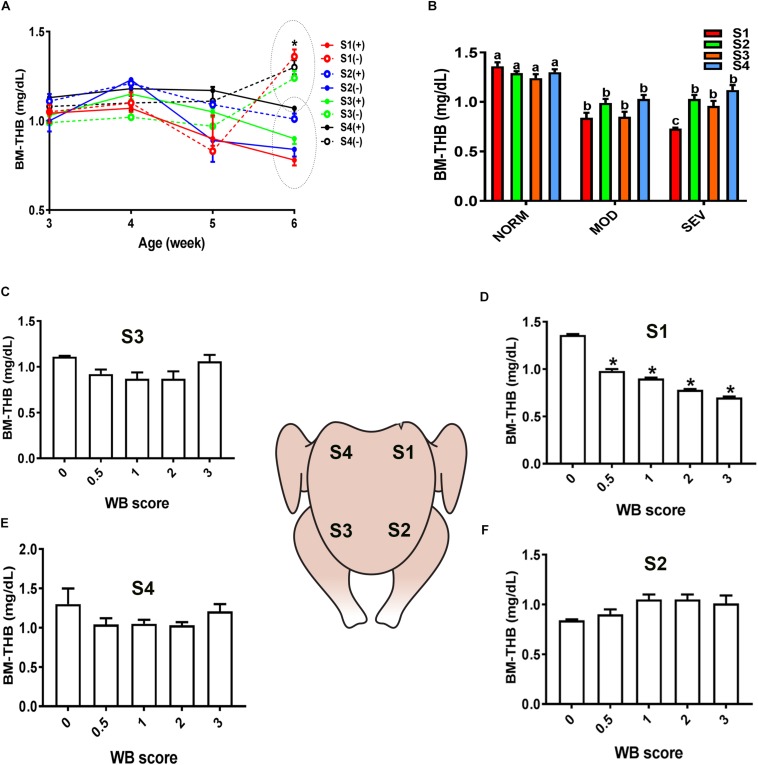
Dysregulation of total hemoglobin (THB) levels in the breast muscle of WB-affected broilers. DRS measurement shows a significant decrease of THB levels in caudal S1 region of breast muscle **(A)**. Decrease of THB levels in MOD and SEV woody breast **(B)**. Decrease of THB levels in WB with score 0.5 in broiler breast muscle in regions S1, S2, S3, and S4 **(C–F)**. Data are presented as mean ± SEM (*n* = 50/group). ^∗^ and different letters indicate significant difference at *P* < 0.05. (+) WB-affected birds, (–) non-affected birds.

**FIGURE 3:**
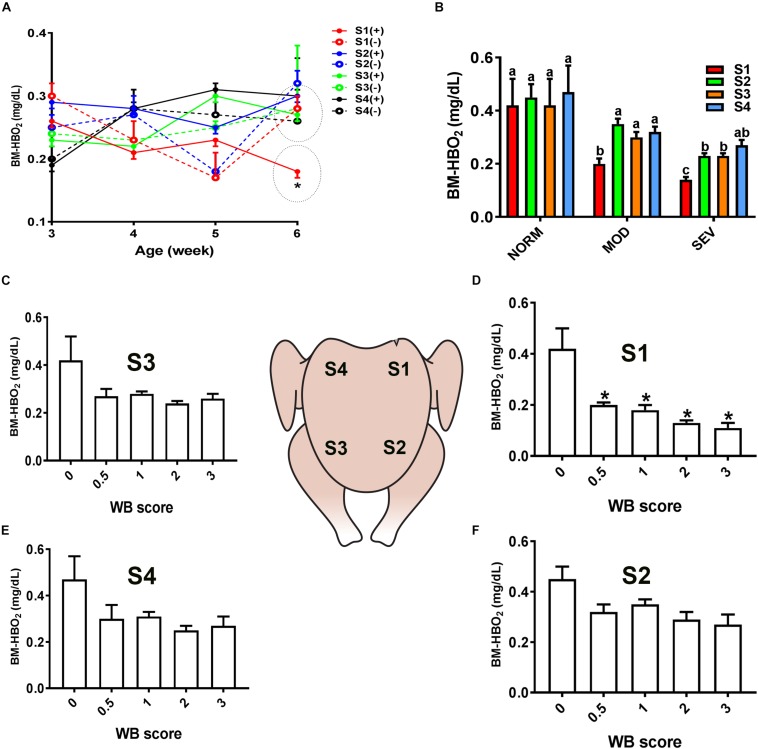
Dysregulation of oxygenated hemoglobin (HBO_2_) levels in the breast muscle of WB-affected broilers. DRS measurement shows a significant decrease of HBO_2_ levels in caudal S1 region of breast muscle **(A)**. Decrease of HBO_2_ levels in MOD and SEV woody breast **(B)**. Decrease of HBO_2_ levels in WB with score 0.5–3 in broiler breast muscle in regions S1, S2, S3, and S4 **(C–F)**. Data are presented as mean ± SEM (*n* = 50/group). ^∗^ and different letters indicate significant difference at *P* < 0.05. (+) WB-affected birds, (–) non-affected birds.

Similarly, evaluation of HB-based parameters showed a similar trend as for sO_2_ levels. As shown in Figures 2, 3, THB and oxygenated HB (HBO_2_) levels were significantly reduced in S1 region of MOD and SEV WB compared to NORM breasts.

Analysis of blood gases and hematology, using iSTAT portable clinical analyzer, showed that sO_2_ (*P* = 0.07), Hct (*P* = 0.06), and HB (*P* < 0.05) levels tended to be lower in chicken with WB compared to healthy counterparts (Table 3). Together these data pointed to highly systemic hypoxia and poorly perfused breast muscle in broilers with WB myopathy.

**TABLE 3:** Blood gases, chemistries, and hematology in healthy and WB-affected broilers^1^.

	**Gases**							**Electrolytes**				**Hemato**	
			
	**pH**	**pCO2**	**pO_2_**	**TCO^2^**	**HCO^3^**	**BE**	**sO_2_**	**Na**	**K**	**iCa**	**Glucose**	**Hct**	**Hgb**
		**(mmHg)**	**(mmHg)**	**(mmol/L)**	**(mmol/L)**	**(mmol/L)**	**(%)**	**(mmol/L)**	**(mmol/L)**	**(mmol/L)**	**(mg/dL)**	**(%)**	**(g/dL)**
Normal	7.44 ± 0.04	38.5 ± 8.3	66.5 ± 7.9	26 ± 3.5	25 ± 3.4	1.8 ± 0.3	89.2 ± 3.1	144.8 ± 4.3	4.4 ± 0.4	1.27 ± 0.09	243 ± 8.1	22.2 ± 2.7	7.6 ± 0.1
WB	7.46 ± 0.03	36.9 ± 4	59.3 ± 5.4	26.3 ± 2.4	25.4 ± 2.1	2.4 ± 2.3	80 ± 4.4	143.1 ± 3.7	4.2 ± 0.3	1.28 ± 0.04	254 ± 3.3	18.1 ± 1.7	6.1 ± 0.2
*P*-value	0.69	0.86	0.46	0.94	0.92	0.79	0.10	0.76	0.69	0.92	0.22	0.21	<0.0001

*^1^Means represent the average response of eight replicate pens/treatment and five to eight birds/pen. pCO_2_, partial pressure of carbon dioxide; pO_2_, partial pressure of oxygen; TCO_2_, total carbon dioxide; HCO_3_, bicarbonate; BE, base excess; sO_2_, oxygen saturation; iCa, ionized calcium; Hct, hematocrit; Hgb, hemoglobin; Na, sodium; K, potassium.*

**FIGURE 4:**
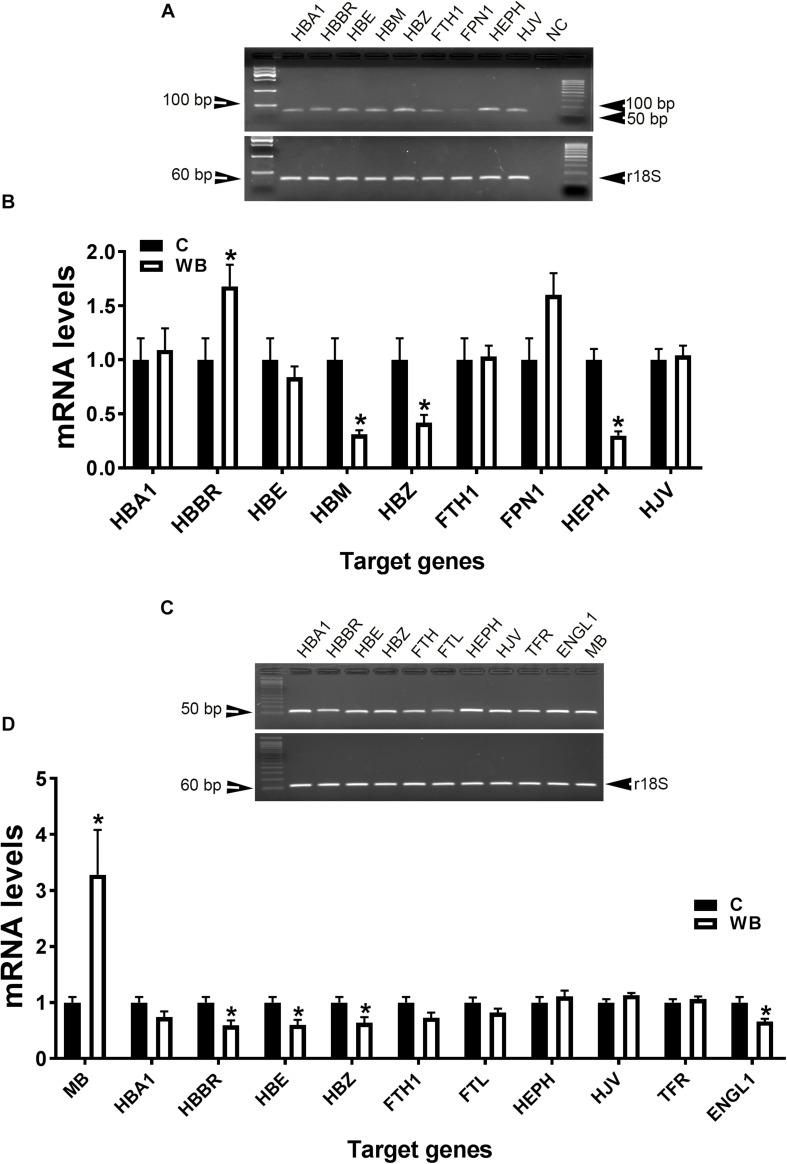
Dysregulation of oxygen-sensing genes in WB-affected broilers. Oxygen-sensing genes are expressed in broiler blood **(A)**, and breast muscle **(C)**. Dysregulation of oxygen-sensing genes in blood **(B)** and breast muscle of WB-affected birds **(D)**. mRNA abundances were determined by qPCR and analyzed by 2^–ΔΔ*Ct*^ method. Data are presented as mean ± SEM (*n* = 8/group). ^∗^ indicates significant difference at *P* < 0.05.

In support of the abovementioned data, molecular analysis showed that blood HB subunit Mu (HBM), Zeta (HBZ), and hephaestin (HEPH) expression were significantly down regulated; however, the expression of the subunit rho of HB beta (HBBR) was upregulated in chicken with WB compared to healthy counterparts (Figures 4A,B). The breast muscle HBBR, HBE, HBZ, and hypoxia-inducible factor prolyl hydroxylase 2 (PHD2 also known as EGLN1) mRNA abundances were significantly down regulated in WB compared to normal birds (Figures 4C,D). However, MB gene expression was significantly upregulated in the breast of WB-affected compared to non-affected birds (Figure 4D).

### HIF-1α and Its Upstream Mediators Are Activated in Chickens With WB Myopathy

**FIGURE 5:**
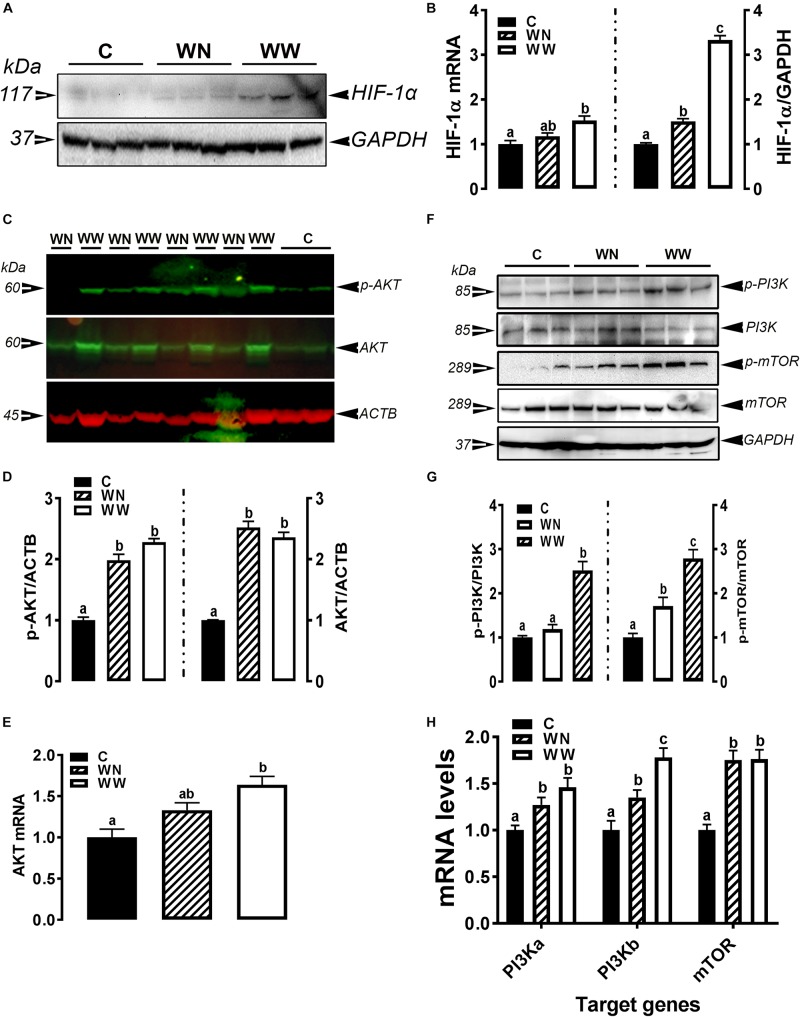
Activation of hypoxia signaling pathway in breast muscle of WB-affected birds. Upregulation of HIF-1α mRNA and protein in WB-affected birds **(A,B)**. Upregulation of HIF-1α upstream mediators including AKT **(C–E)**, and PI3K-mTOR **(F–H)**. Protein expression was measured by conventional and fluorescent western blot, and relative gene expression was determined by qPCR. Data are presented as mean ± SEM (*n* = 8/group). Different letters indicate significant difference at *P* < 0.05. Western blot image is a representative of three replicates.

As illustrated in Figures 5A,B, the expression of HIF-1α at mRNA and protein levels was significantly induced in breasts (affected caudal area, WW and apparent healthy area, WN) of broilers with WB myopathy compared to their healthy counterparts, indicating a hypoxic status. The phosphorylated levels of AKT at Ser473 site, mTOR at Ser2481 site, and PI3K P85 at Tyr458 site, as well as their mRNA levels were significantly increased in breasts (affected caudal area, WW and apparent healthy area, WN) of broilers with WB myopathy compared to their healthy counterparts (Figures 5C–H).

### Plasma *Myo*-Inositol and Metabolite Levels and Breast Muscle Mineral Profiles in WB-Affected and Unaffected Birds

Plasma glucose, cholesterol, triglyceride, total proteins, CK, NEFA, and MI did not differ between WB-affected and unaffected birds (Table 4). The concentrations of Ca, Na, and Zn were significantly higher in the breast muscle of WB-affected broilers compared to their healthy counterparts (Table 4). However, the levels of the elements K, Mg, P, and S were significantly lower in WB-affected compared to unaffected group (Table 4). The levels of Al, Cu, Fe, and Mn remain unchanged between the two groups (Table 4).

**TABLE 4:** Plasma metabolite and myoinositol levels and breast muscle mineral profile in healthy and WB-affected birds^1^.

**Parameters^2^**	**Animal status**	
	
	**C**	**WB**
**Plasma metabolites**		
Glucose (mg/dL)	243.3 ± 8.6	254.3 ± 3.3
Cholesterol (mg/dL)	104.8 ± 5.7	110.1 ± 2.2
Triglycerides (mg/dL)	27.87 ± 2.2	34.42 ± 3.2
Total proteins (g/dL)	28.83 ± 1.7	29.71 ± 1.7
CK (10^3^ U/L)	68.1 ± 10.4	93.37 ± 11
NEFA (mmol/L)	0.24 ± 0.01	0.28 ± 0.02
*Myo*-inositol (μM)	268.85 ± 19.5	318.39 ± 21
**Muscle minerals (ppm)**		
Al	9.0 ± 0.1	9.0 ± 0.1
Ca	46.8 ± 3.0	71.3 ± 1.5^∗^
Cu	0.7 ± 0.04	0.7 ± 0.04
Fe	9.9 ± 1.5	9.5 ± 0.5
K	2,960 ± 51	2,421 ± 17^∗^
Mg	280.7 ± 5.6	174.6 ± 4.4^∗^
Mn	5.0 ± 0.07	5.0 ± 0.04
Na	267 ± 11.1	705.7 ± 29^∗^
P	2,222 ± 38	1,561 ± 27^∗^
S	1,949 ± 27	1,601 ± 29^∗^
Zn	6.4 ± 0.3	12.1 ± 0.5^∗^

*^1^Means represent the average response of eight replicate pens/treatment and five to eight birds/pen. ^∗^*P* < 0.05. ^2^CK, creatine kinase; Al, aluminum; Ca, calcium; Cu, copper; Fe, iron; K, potassium; Mg, magnesium; Mn, manganese; Na, sodium; P, phosphorus; S, sulfur; Zn, zinc.*

### Quantum Blue Reduces WB Severity via Modulation of Oxygen-Sensing Genes

**FIGURE 6:**
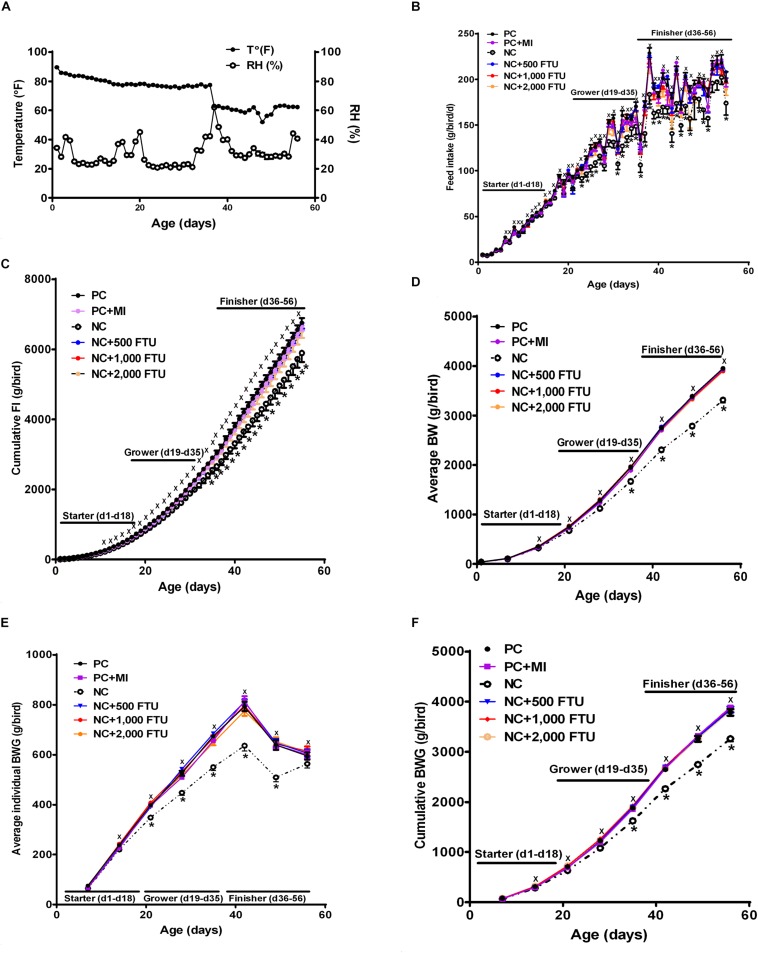
Effect of QB-enriched diets on broiler growth performances. **(A)** Environmental condition (RH and T°) of the barn. QB did not affect individual and cumulative feed intake **(B,C)**, and average BW and BWG **(D–F)**. Data are presented as mean ± SEM (*n* = 96 birds/group). ^∗^ indicates significant difference at *P* < 0.05.

**FIGURE 7:**
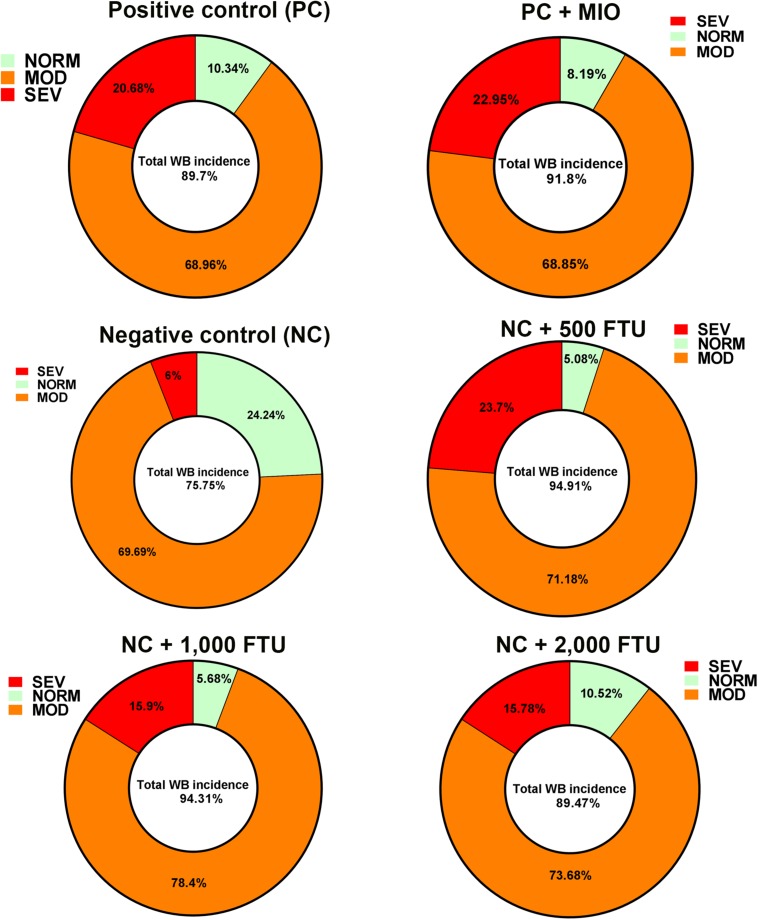
QB-enriched diets reduces the severity of WB incidence. At day 56 and after slaughter process, breast filets were macroscopically scored and classified to WB categories to normal (NORM, score 0), moderate (MOD, score 0.5–1.5), and severe (SEV, score 2–3).

In attempt to identify a nutritional strategy to reduce WB incidence, we used different increasing doses of QB. Birds were maintained under standard environmental conditions (Figure 6A) and QB was supplemented at 500; 1,000; and 2,000 FTU/kg diet for 56 days. As shown in Figures 6B–F and as expected, NC birds (Ca- and P-deficient diet) decreased their individual and cumulative FI, and in turn, showed lower average body weight and body weight gain compared to standard and PC diet as well as to QB-supplemented diets. Although the activity rate recovery of QB was as expected (Table 5), QB did not have any significant effect on FCR and FE (Table 6). However, QB supplementation quadratically increased (*P* < 0.05) hot and cold carcass weight, breast meat yield, and wing and leg yield (Table 7). Although the incidence of WB myopathy did not differ between the PC and QB-fed groups, high dose (1,000 and 2,000 FTU) of QB significantly reduced the severity of WB by ∼5% compared to the PC (Figure 7).

**TABLE 5:** Phytase activity (FTU/kg) recovered in the experimental diets.

**Experimental diet**	**Starter phase**	**Grower phase**	**Finisher phase**
Positive control (PC)	<50	<50	<50
PC + 0.30% inositol	<50	<50	<50
Negative control (NC)	<50	<50	<50
NC + 500 FTU	385	840	550
NC + 1,000 FTU	834	1,480	1,310
NC + 2,000 FTU	1,850	2,490	1,950

*^1^The phytase used was Quantum Blue (AB Vista, Marlborough, United Kingdom) with an expected activity of 5,000 FTU/g.*

**TABLE 6:** Effects of QB on growth performances^1^.

**Diet**	**FCR**	**FE**	**Mortality (%)**
Positive control (PC)	1.7106	0.5845	7.3
PC + myo-inositol (MI)	1.6914	0.5912	2.6
Negative control (NC)	1.7786	0.5622	1.4
NC + 500 FTU/kg phytase	1.6976	0.5890	3.1
NC + 1,000 FTU/kg phytase	1.7247	0.5797	4
NC + 2,000 FTU/kg phytase	1.7005	0.5880	7.3

*^1^Means represent the average response of eight replicate pens/treatment and 20 birds/pen. FCR, feed conversion ratio; FE, feed efficiency.*

**TABLE 7:** Live weight and carcass and cut up weight of broilers fed myo-inositol or phytase from hatch to 56-days post-hatch^1^.

**Diet**	**Live weight (g)**	**Hot carcass weight (g)**	**Cold carcass weight (g)**	**Breast meat weight (g)**	**Wing weight (g)**	**Tender weight (g)**	**Leg weight (g)**	**Rack weight (g)**
Positive control (PC)	3,970	3,018	3,065	886	293	177	921	770
PC + myo-inositol (MI)	3,949	3,006	3,057	872	293	172	926	777
Negative control (NC)	3,313	2,507	2,451	689	259	144	791	643
NC + 500 FTU/kg phytase	3,950	3,022	3,078	917	294	178	911	763
NC + 1,000 FTU/kg phytase	3,928	3,009	3,046	898	294	175	915	753
NC + 2,000 FTU/kg phytase	3,875	2,957	3,015	877	291	174	921	744
**Pooled SEM**								
Diet *P*-value	0.0001	0.0001	0.0001	0.0001	0.0001	0.0001	0.0001	0.0001
**Contrast *P*-value^2^**								
PC vs. NC	*P* < 0.01	*P* < 0.01	*P* < 0.01	*P* < 0.01	*P* < 0.01	*P* < 0.01	*P* < 0.01	*P* < 0.01
PC vs. MI	NS	NS	NS	NS	NS	NS	NS	NS
Linear phytase	*P* < 0.05	*P* < 0.05	*P* < 0.01	*P* < 0.05	*P* < 0.05	*P* < 0.05	*P* < 0.01	*P* < 0.01
Quadratic phytase	*P* < 0.05	*P* < 0.05	*P* < 0.01	*P* < 0.01	*P* < 0.05	*P* < 0.05	NS	*P* < 0.01
MI vs. NC + 2,000 FTU/kg	NS	NS	NS	NS	NS	NS	NS	NS

*^1^Means represent the average response of eight replicate pens/treatment and five to eight birds/pen. ^2^Non-orthogonal contrast statements were adjusted using *post hoc* Scheffe’s test for significance (Kaps and Lamberson, 2004). NS, non-significant (*P* > 0.05).*

**FIGURE 8:**
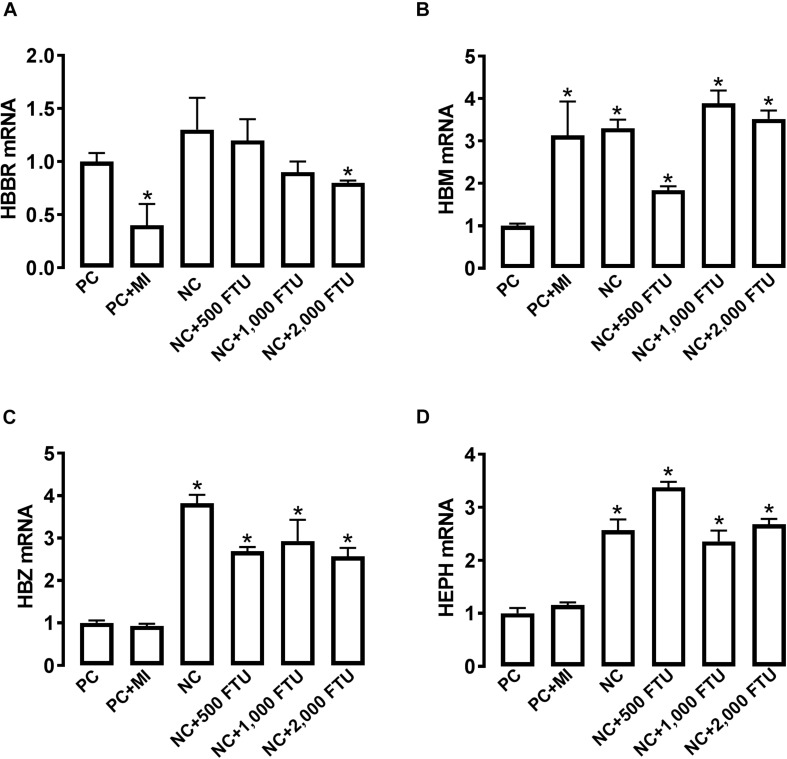
QB-enriched diets modulate the expression of oxygen-sensing genes in broiler blood. Relative gene expression of HBBR **(A)**, HBM **(B)**, HBZ **(C)**, and HEPH **(D)** was determined by qPCR and analyzed by 2^–ΔΔ*Ct*^ method using PC group as a calibrator. Data are presented as mean ± SEM (*n* = 8 birds/group). ^∗^ indicates significant difference at *P* < 0.05 compared to PC group. HBBR, hemoglobin subunit rho; HBM, hemoglobin subunit mu; HBZ, hemoglobin subunit zeta; HEPH, hephaestin.

**FIGURE 9:**
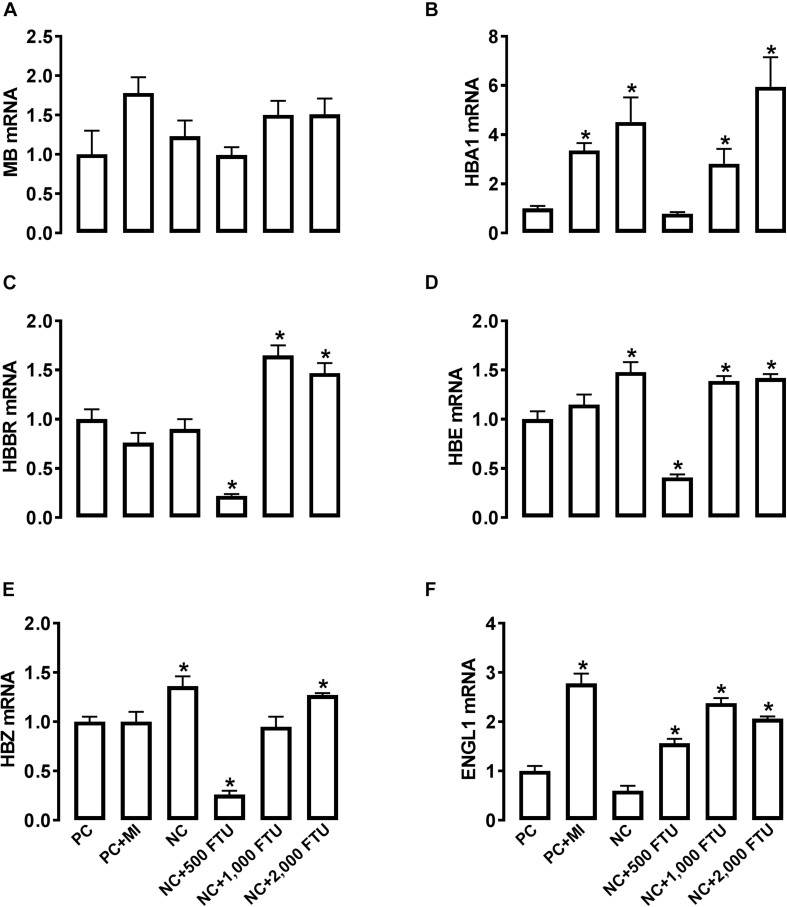
QB-enriched diets modulate the expression of oxygen-sensing genes in broiler breast muscle. Relative gene expression of MB **(A)**, HBA1 **(B)**, HBBR **(C)**, HBE **(D)**, HBZ **(E)**, and ENGL1 **(F)** was determined by qPCR and analyzed by 2^–ΔΔ*Ct*^ method using PC group as a calibrator. Data are presented as mean ± SEM (*n* = 8 birds/group). ^∗^ indicates significant difference at *P* < 0.05 compared to PC group. ENGL1, Egl nine homolog 1; HBA1, hemoglobin alpha 1; HBBR, hemoglobin subunit rho; HBE, hemoglobin E; HBZ, hemoglobin subunit zeta; MB, myoglobin.

At molecular levels, QB supplementation reverses the expression profile of oxygen homeostasis-related genes; i.e., significant down regulation of HBBR (at 2,000 FTU) and upregulation of HBM, HBZ, and HEPH (all doses of QB) in blood (Figures 8A–D), as well as a significant upregulation of HBA1, HBBR, HBE, HBZ, and EGLN1 in breast muscle compared to the PC with the doses 1,000 and 2,000 FTU are the most efficient (Figures 9A–F).

At systemic levels, QB supplementation did not elicit any change to the plasma metabolite levels in healthy chickens, except a reduction of CK concentrations with QB superdose (2,000 FTU). At tissue levels, QB-enriched diets reduce Cu and Fe levels. However only1,000 FTU of QB reduces Ca levels in breast muscle compared to the PC-fed group (Table 8). QB supplementation slightly increases MI levels in the breast muscle of unaffected chickens (Table 8).

**TABLE 8:** Plasma metabolite and myo-inositol levels and breast muscle myo-inostol and mineral concentrations in healthy chickens^1^.

**Parameters^2^**	**Diets**					
	
	**PC**	**PC + MIO**	**NC**	**NC + 500 FTU**	**NC + 1,000 FTU**	**NC + 2,000 FTU**
**Plasma metabolites**						
Glucose (mg/dL)	254.5 ± 5	251.8 ± 5	263.5 ± 5	244.2 ± 3	244.5 ± 6	257.5 ± 8
Cholesterol (mg/dL)	109.1 ± 2	105.2 ± 5	114.5 ± 6	119.4 ± 4	104.7 ± 3	106.0 ± 1.8
Triglycerides (mg/dL)	27.8 ± 2	34.4 ± 3.2	29.3 ± 3.6	26.6 ± 2	32.2 ± 3	26.6 ± 2
Total proteins (g/dL)	3.3 ± 0.15	3.4 ± 0.15	3.5 ± 0.20	3.5 ± 0.1	3.6 ± 0.12	3.4 ± 0.13
CK (10^3^ U/L	102 ± 17	79.4 ± 12.8	25.3 ± 5.7^∗^	125 ± 32	90.6 ± 7.4	77.41 ± 12
NEFA (mmol/L)	0.2 ± 0.01	0.25 ± 0.02	0.25 ± 0.04	0.2 ± 0.01	0.24 ± 0.01	0.22 ± 0.01
*Myo*-inositol (μM)	284 ± 22	260 ± 31	320 ± 35	334 ± 36	260 ± 39	266 ± 42
**Muscle minerals (ppm)**						
Al	9.0 ± 0.5	9.5 ± 0.1	–	8.8 ± 0.1	8.6 ± 0.1	8.9 ± 0.1
Ca	52.9 ± 6.7	39.9 ± 3.1	–	40.8 ± 2.3	35.2 ± 0.2^∗^	63.9 ± 11
Cu	0.73 ± 0.1	0.43 ± 0.1	–	0.42 ± 0.05^∗^	0.40 ± 0.1^∗^	0.34 ± 0.1^∗^
Fe	12 ± 1.3	18.3 ± 7.2	–	6.7 ± 0.76^∗^	5.2 ± 0.28^∗^	6.5 ± 0.71^∗^
K	3041 ± 81	2897 ± 142	–	3207 ± 185	2976 ± 20	2746 ± 116
Mg	292 ± 6.2	272 ± 14.5	–	288 ± 26.4	289 ± 5.7	264 ± 15.7
Mn	4.99 ± 0.2	5.09 ± 0.2	–	4.93 ± 0.06	4.99 ± 0.04	5.17 ± 0.3
Na	263 ± 19	252 ± 21.2	–	250 ± 30.2	253 ± 12.4	313 ± 46.1
P	2326 ± 35	2163 ± 109	–	2316 ± 164	2232 ± 31	2097 ± 106
S	1993 ± 20	1844 ± 69	–	2094 ± 114	1937 ± 41	1917 ± 51
Zn	7.8 ± 1.2	5.7 ± 0.3	–	7.3 ± 0.9	5.7 ± 0.3	5.84 ± 0.2
**Muscle *Myo*-inositol**						
*Myo*-inositol (nmol/g wt)	512 ± 26	688 ± 31	512 ± 15	510 ± 20	509 ± 31	602 ± 35

*^1^Means represent the average response of eight replicate pens/treatment and five to eight birds/pen. ^∗^A significant difference from the control (PC) group at *P* < 0.05. ^2^CK, creatine kinase; Al, aluminum; Ca, calcium; Cu, copper; Fe, iron; K, potassium; Mg, magnesium; Mn, manganese; Na, sodium; P, phosphorus; S, sulfur; Zn, zinc.*

## Discussion

The signaling pathways and molecular mechanisms involved in WB myopathy, which is an emerging challenge to the poultry industry worldwide, remain largely undefined. Here, using a combination of the DRS technique and the portable clinical analyzer iSTAT system, we showed a systemic hypoxic status and a poorly oxygenated breast muscle in broilers with WB myopathy compared to their healthy counterparts.

The DRS has been used in several studies to measure tissue scattering, THB content, and vascular oxygenation (Vishwanath et al., 2009; Dhar et al., 2012; Dadgar et al., 2018). The DRS-based measurement of broiler breast muscle oxygenation status can provide a non-destructive and non-invasive tool for an early detection of WB-susceptible birds and, thereby, could aid in the selection of appropriate prevention/intervention strategy.

Similarly, the iSTAT system is gaining popularity in biological research for blood analysis and has been validated on a wide range of species including birds (Schaal et al., 2016), reptiles (Harms et al., 2003), fish (Harter et al., 2014), and mammals (Sediame et al., 1999; Stockard et al., 2007). Although the specific type of hypoxia is not known at this time point, both DRS- and iSTAT-based measurement suggested a complex hypoxia. Indeed, the low oxygen levels in the circulation and in breast muscle of WB birds indicate both a circulatory and a hypoxemic hypoxia (anoxia) (Fedorova, 1964). The low levels of HB in the circulation indicate a potential anemic hypoxia (Cain and Chapler, 1988) which results in a reduced ability of the blood to carry oxygen and, thereby, a diminished supply of oxygen to the breast muscle. A metabolic hypoxia, which might due to high demand for oxygen by the breast muscle that exceed the supply/delivery, is not ruled out (Chappell et al., 2019).

Whatever the type of hypoxia, it is evident that circulatory and breast muscle oxygen homeostasis are altered in birds with WB myopathy. This is supported by the dysregulation of oxygen transport-related molecules including HB subunits (mu, HBM and zeta, HBZ) in red blood cells, and myoglobin (MB), HBBR (epsilon HBE), and HBZ in breast muscle of WB-affected birds compared to their healthy counterparts. The major oxygen-transport proteins in vertebrate blood are HBs and hemerythrins with iron as the prosthetic group. These metalated and multi-subunit proteins are responsible primarily for the sensing, transport, and/or storage of oxygen (Terwilliger, 1998).

Until recently, it has been thought that vertebrate HB is expressed only in erythrocytes. Here we found that HB subunits are expressed not only in red blood cells but also in breast muscle corroborating previous studies that have reported HB expression in a wide variety of non-erythroid cells and tissues including neurons (Ohyagi et al., 1994; Biagioli et al., 2009; Schelshorn et al., 2009), macrophage (Liu et al., 1999), eye lens (Wride et al., 2003), and breast cancer cells (Gorr et al., 2011). The upregulated expression of HBBR in blood, MB in breast, and down regulation of the other subunits (HBM and HBZ) in both blood and breast muscle of WB birds indicated that these subunits have different oxygen affinities or response to allosteric modifiers (Terwilliger, 1998). Together, the low oxygen levels combined with the dysregulation of oxygen-sensing genes indicate a hypoxic status in the breast muscle of WB-affected birds (Gorr et al., 2004; Grek et al., 2011; Xia et al., 2016; Cadiz et al., 2017).

To gain further insights in the etiology of this myopathy and its underlying molecular mechanism, we assess the hypoxia signaling interactive pathway. The upregulation of HIF-1α and down regulation of PHD2 (also known as EGNL1) expression in the breast muscle of WB-affected birds supported the DRS and iSTAT data and confirmed the hypoxic status. Central to the molecular mechanisms underlying oxygen homeostasis are HIF-1α and HIF-2α that function as master regulators of the adaptive response to hypoxia (Nakazawa et al., 2016). HIFs form a heterodimer consisting of a constitutively expressed HIF-1β subunit and oxygen-regulated α subunits (HIF-1α or HIF-2α) (Majmundar et al., 2010; Keith et al., 2011). A HIF-3α has been also described (Ema et al., 1997). Under normoxic conditions, HIF α-subunits are hydroxylated by PHDs (also known as HIF-1 prolyl hydroxylases HPH or EGLN1) and targeted for proteasomal degradation by the Von Hippel–Lindau disease tumor suppressor protein (pVHL), a component of the E3 ubiquitin ligase complex (Lee et al., 2016). These PHDs are 2-OG-dependent dioxygenase enzymes which require oxygen for their hydroxylation action, and hence they are inactivated when the oxygen level is insufficient, and in turn, enhances the activity of HIF by stabilizing its α subunit (Epstein et al., 2001).

In agreement with previous studies (Gingras et al., 2001; Jiang et al., 2001), the activation of phosphatidyl inositol-4,5-bisphosphate-3-kinase (PI3K) – protein kinase B (PKB or AKT) – mechanistic target of rapamycin (mTOR) pathway in our experimental conditions indicates that this pathway might upregulate HIF-1α protein translation. PI3K regulates protein syntheses through its target AKT and downstream component mTOR. mTOR mediates its action via phosphorylation of the eukaryotic translation initiation factor 4E(eIF-4E) binding protein (4E-BP1) disrupting the integrity of these two components, which is essential for inhibiting cap-dependent mRNA translation, resulting in enhanced HIF-1α protein translation (Treins et al., 2002). Land and Tee (2007) have shown that Rheb-specific activation of mTOR enhanced the transcriptional activity of HIF-1α during hypoxia. It has also been reported that mTOR shuttles between the cytoplasm and the nucleus and that this cytoplasmic–nuclear interchange of mTOR is necessary for the mTOR-dependent phosphorylation of S6K1p70 S6 kinase (S6K) which, in turn, induces HIF-1α protein translation (Kim and Chen, 2000; Kim et al., 2014).

Intriguingly, we found that HEPH gene expression was down regulated in the circulation but not in breast muscle of WB birds. Currently, HEPH is well known to be involved in the intestinal metabolism of iron and possibly copper (Chen et al., 2006). It is a transmembrane copper-dependent ferroxidase responsible for transporting dietary iron from intestinal enterocytes into the circulation system and mediates iron efflux in cooperation with the basolateral iron transporter, ferroportin 1 (FPN1) which is slightly upregulated in blood of WB birds. However, copper and iron levels in the breast muscle did not differ between WB-affected and unaffected birds. This suggests that HEPH may have other roles in the circulation that need to be defined. As it belongs to the same family as ceruloplasmin, it is possible that HEPH is involved in copper/iron detoxification. Interestingly and similar to dog hereditary muscle dystrophy (Mehta et al., 1989), we found a differential mineral element profile; increased levels of Ca, Na, and Zn, and decreased levels of K, Mg, P, and S in beast muscle of WB birds. Although a mechanistic interaction between minerals and WB myopathy is lacking, our data suggest that WB might be associated with mineral overload/deficiency. It has been shown that hypoxia increases intracellular Zn levels (Bernal et al., 2008) and intracellular Zn overload has been reported to alter skeletal muscle contractility (Isaacson and Sandow, 1963; Bernal et al., 2011). Hypoxia was also found to increase basal Ca and Na concentrations, and reduce K and P levels (Weiss et al., 1989; Yadav et al., 2013; Shi et al., 2014). It is clear from several lines of evidence that defect in intracellular element (Ca, Na, P, K, etc.) homeostasis is a hallmark of muscular dystrophies (Altamirano et al., 2012; Weber et al., 2012; Mijares et al., 2014; Bkaily and Jacques, 2017; Saito et al., 2017). Although further in-depth mechanistic studies are warranted, it is possible that hypoxia-induced intracellular mineral unbalance alters muscle ATP concentration and energy utilization, which activates the master energy sensor AMPK (data not shown) and, in turn, leads to reactive oxygen species (ROS) production, inflammation, and muscle fiber degeneration (Irrcher et al., 2009; Guo et al., 2014).

Because QB has been reported to improve hematological parameters (number of red blood cells, HB, and Hct) in channel catfish (Peatman and Beck, 2016; Ferreira and Aurélio Lopes Della Flora, 2017), we hypothesized that QB might reduce WB incidence. Although the total incidence of WB did not differ between all groups, QB reduces the severity of WB by ∼5% compared to the control group. Ameliorating WB severity is very critical and beneficial not only for the animal well-being but also for the poultry industry and the consumer because the severity of the myopathy can adversely affect consumer perception and acceptance of raw cut up parts and/or quality for further processed meat products (Kuttappan et al., 2017), resulting in significant economic loss to the industry. The effect of QB seemed to be mediated via the increased expression of oxygen-sensing genes leading to enhanced oxygenation in both blood and breast muscle. QB is a phosphatase enzyme that catalyzes the hydrolysis of phytate, thereby liberating utilizable inorganic phosphate and MI. MI has been shown to increase oxygen pressure and antagonize the hypoxic setting (Derbal-Wolfrom et al., 2013). Although the mode of action of QB merits further investigations, it is possible that QB also improve mineral and nutrient uptake by destroying phytate and its other downstream hydrolysis products.

## Conclusion

In conclusion, this is the first mechanistic evidence, to our knowledge, showing that WB myopathy is associated with systemic and local breast muscle hypoxia, and we identified a potential nutritional strategy to reduce its severity.

## Data Availability Statement

All datasets generated for this study are included in the manuscript/supplementary files.

## Ethics Statement

The present study was conducted in accordance with the recommendations in the guide for the care and use of laboratory animals of the National Institutes of Health and the protocols were approved by the University of Arkansas Animal Care and Use Committee under protocol No. 16084.

## Author Contributions

SaD conceived and designed the study. EG and JF conducted the experiments, determined gene and protein expression, and analyzed the data. SiD, AD, and NR measured the oxygen levels using the DRS technique. BM and SO determined the WB incidence by palpation and scoring. CL, HW, and CB measured the MI and determined AKT expression by the fluorescent western blot. CW provided the QB. SaD wrote the manuscript with a critical review by CW, MK, NR, and SR.

## Conflict of Interest

CW was employed by company AB Vista. The remaining authors declare that the research was conducted in the absence of any commercial or financial relationships that could be construed as a potential conflict of interest.

## Footnotes

1 https://omlc.org/spectra/hemoglobin/
